# Supplementary material for: Long-term outcomes of hospitalized patients with SARS-CoV-2/COVID-19 with and without neurological involvement: 3-year follow-up assessment
Source: PLoS Med. 2024 Apr 4;21(4):e1004263. doi: 10.1371/journal.pmed.1004263 (PMC10994395; doi:10.1371/journal.pmed.1004263)
Supplement: S1 Table — (DOCX) [file pmed.1004263.s002.docx]

**Supplemental Table 1. Proof of propensity match between neurological and control cohorts.** Mean±SD. Two-tailed t-test used to compare continuous variables. *Abbreviations: SD (standard deviation).*

|  | **Neurological Cohort**  **(N=414)** | **Control Cohort**  **(N=1199)** | **P-value** |
| --- | --- | --- | --- |
| **Patient Characteristics** |  |  |  |
| Age, years old (at admission) | 69.71±15.80 | 70.26±15.14 | 0.539 |
| COVID-19 Severity Score | 3.53±2.07 | 3.53±2.10 | 0.996 |
