# Supplementary material for: Long-term outcomes of hospitalized patients with SARS-CoV-2/COVID-19 with and without neurological involvement: 3-year follow-up assessment
Source: PLoS Med. 2024 Apr 4;21(4):e1004263. doi: 10.1371/journal.pmed.1004263 (PMC10994395; doi:10.1371/journal.pmed.1004263)
Supplement: S2 Table — (DOCX) [file pmed.1004263.s003.docx]

**Supplemental Table 2**. **Laboratory values of patients in the neurological and control cohorts at COVID-19 hospitalization admission and most recent follow-up.** Mean±SD. χ^2^ used to compare categorical variables and two-tailed t-test use to compare continuous variables between groups. *Abbreviations: SD (standard deviation); SBP (systolic blood pressure); DDIM (D-dimer); INR (international normalized ratio); BUN (blood urea nitrogen); Cr (creatinine); AST (aspartate aminotransferase); ALT (alanine aminotransferase); WBC (white blood cell); lymph (lymphocyte); FERR (ferritin); CRP (C-reactive protein); LDH (lactate dehydrogenase); BNP (brain natriuretic peptide); TNT (troponin-T).*

|  | Neurological Cohort  (N=414) | | | Control Cohort  (N=1199) | | | Intercohort P-Values | |
| --- | --- | --- | --- | --- | --- | --- | --- | --- |
| **Lab Values** | **Admission** | **Follow-Up** | **P-val** | **Admission** | **Follow-Up** | **P-val** | **Admission** | **Follow-Up** |
| Temperature [^o^F] | 98.86±1.22 | 98.22±0.74 | **<0.001** | 99.05±1.33 | 98.11±2.05 | **<0.001** | **0.004** | 0.057 |
| SBP [mmHg] | 133.64±27.46 | 124.19±24.44 | **<0.001** | 135.09±25.25 | 127.24±22.49 | **<0.001** | 0.173 | **0.013** |
| Mean arterial pressure [mmHg] | 122.38±83.16 | 116.77±61.21 | 0.135 | 103.33±65.24 | 111.56±63.52 | **<0.001** | **<0.001** | 0.070 |
| DDIM [ug/mL FEU] | 4.27±5.57 | 3.17±4.42 | **<0.001** | 3.46±4.84 | 3.05±14.05 | 0.170 | **0.004** | 0.397 |
| INR [seconds] | 15.75±7.00 | 15.48±4.48 | 0.254 | 15.32±6.82 | 15.38±4.99 | 0.403 | 0.139 | 0.352 |
| BUN [mg/dL] | 35.35±35.08 | 26.26±24.06 | **<0.001** | 29.94±28.38 | 22.64±20.52 | **<0.001** | **0.002** | **0.003** |
| Cr [mg/dL] | 2.15±2.74^ | 1.79±2.27 | **0.020** | 1.90±2.43 | 1.57±1.90 | **<0.001** | 0.050 | **0.039** |
| Sodium | 138.58±8.07 | 139.18±4.85 | 0.098 | 137.75±7.48 | 139.10±4.30 | **<0.001** | 0.033 | 0.383 |
| Glucose [mg/dL] | 190.21±179.35 | 136.47±74.92 | **<0.001** | 172.68±135.56 | 130.12±62.67 | **<0.001** | **0.035** | 0.061 |
| AST [U/L] | 48.76±53.08 | 61.20±257.39 | 0.168 | 56.87±105.55 | 52.89±361.85 | 0.357 | **0.022** | 0.306 |
| ALT [U/L] | 33.8544.55 | 51.74±267.41 | 0.090 | 38.82±71.60 | 36.96±102.81 | 0.304 | 0.050 | 0.137 |
| WBC [k/uL] | 8.13±4.67 | 7.95±3.99 | 0.276 | 8.47±7.31 | 7.91±3.66 | **0.009** | 0.138 | 0.429 |
| Lymph [k/uL] | 1.16±0.76 | 1.67±0.93 | **<0.001** | 1.38±4.94 | 1.74±1.09 | **0.007** | 0.068 | 0.104 |
| FERR [ng/mL] | 1251.76±1550.73 | 964.16±2385.43 | **0.020** | 1261.31±2092.20 | 948.88±1532.12 | **<0.001** | 0.461 | 0.452 |
| CRP [mg/L] | 9.61±9.33 | 5.91±6.94 | **<0.001** | 11.10±9.66 | 5.54±6.27 | **<0.001** | **0.003** | 0.169 |
| Procalcitonin [ug/L] | 1.99±6.17 | 2.23±7.15 | 0.303 | 1.25±4.82 | 1.43±6.10 | 0.211 | **0.013** | **0.021** |
| LDH [U/L] | 411.72±300.88 | 345.73±156.67 | **<0.001** | 404.41±231.39 | 341.15±267.74 | **<0.001** | 0.326 | 0.337 |
| BNP [pg/mL] | 2519.11±4491.13 | 1642.03±3577.47 | **<0.001** | 1825.85±3716.69 | 1242.17±2948.39 | **<0.001** | **0.002** | **0.021** |
| TNT [ug/L] | 0.06±0.19 | 0.26±2.13 | **0.029** | 0.04±0.09 | 0.18±1.53 | **<0.001** | **0.020** | 0.241 |
| Pulse Oximetry [% Oxygenation] | 95.20±5.51 | 97.10±6.31 | **<0.001** | 93.80±7.15 | 97.12±5.56 | **<0.001** | **<0.001** | 0.477 |
