# Supplementary material for: Long-term outcomes of hospitalized patients with SARS-CoV-2/COVID-19 with and without neurological involvement: 3-year follow-up assessment
Source: PLoS Med. 2024 Apr 4;21(4):e1004263. doi: 10.1371/journal.pmed.1004263 (PMC10994395; doi:10.1371/journal.pmed.1004263)
Supplement: S3 Table — (DOCX) [file pmed.1004263.s004.docx]

**Supplemental Table 3. Number of patients treated in the ICU or placed on IMV during hospitalization for each outcome.** Reported as N for both the neurological and control cohorts. Abbreviations: *ICU (intensive care unit); IMV (invasive mechanical ventilation), MACE (major adverse cardiac event).*

|  | **Neurological Cohort (N=414)** | | **Control Cohort (N=1043)** | |
| --- | --- | --- | --- | --- |
|  | ICU (N=30) | IMV (N=24) | ICU (N=77) | IMV (N=59) |
| **Outcomes (Jan 10, 2023)** | | | | |
| Hospital Readmission | 16 | 14 | 46 | 38 |
| Stroke | 1 | 1 | 0 | 0 |
| Heart Attack | 0 | 0 | 4 | 4 |
| MACE | 5 | 3 | 15 | 13 |
| Mortality after discharge |  |  |  |  |
| 0.5 years | 2 | 4 | 2 | 3 |
| 1.0 years | 3 | 4 | 3 | 3 |
| 3.0 years | 4 | 4 | 7 | 5 |
| SARS-CoV-2 reinfection | 0 | 0 | 0 | 1 |
