# Supplementary figures and images for: Long-term outcomes of hospitalized patients with SARS-CoV-2/COVID-19 with and without neurological involvement: 3-year follow-up assessment
Source: PLoS Med. 2024 Apr 4;21(4):e1004263. doi: 10.1371/journal.pmed.1004263 (PMC10994395; doi:10.1371/journal.pmed.1004263)

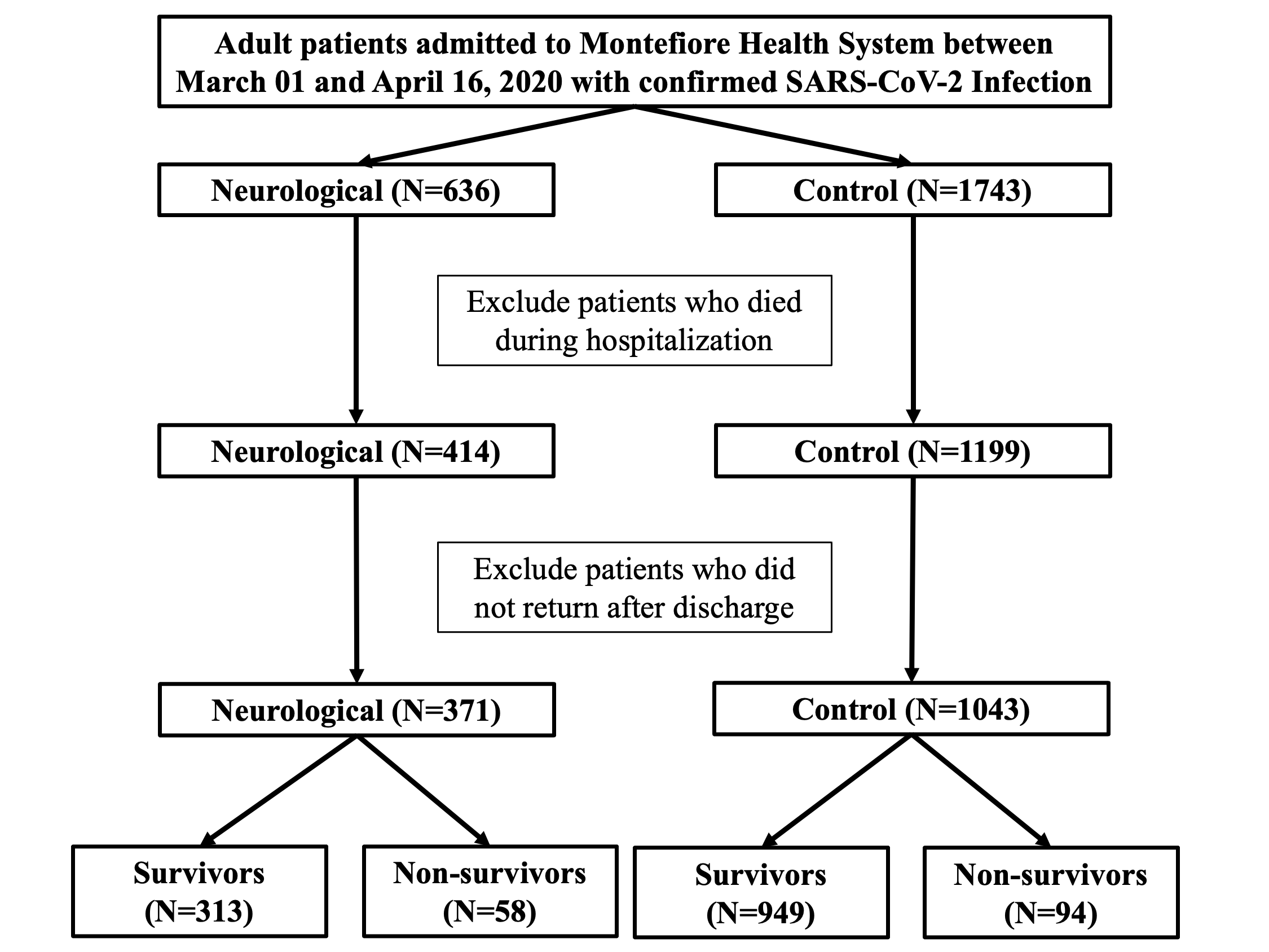

Supplement: S1 Fig — (TIFF) [file pmed.1004263.s005.tiff]

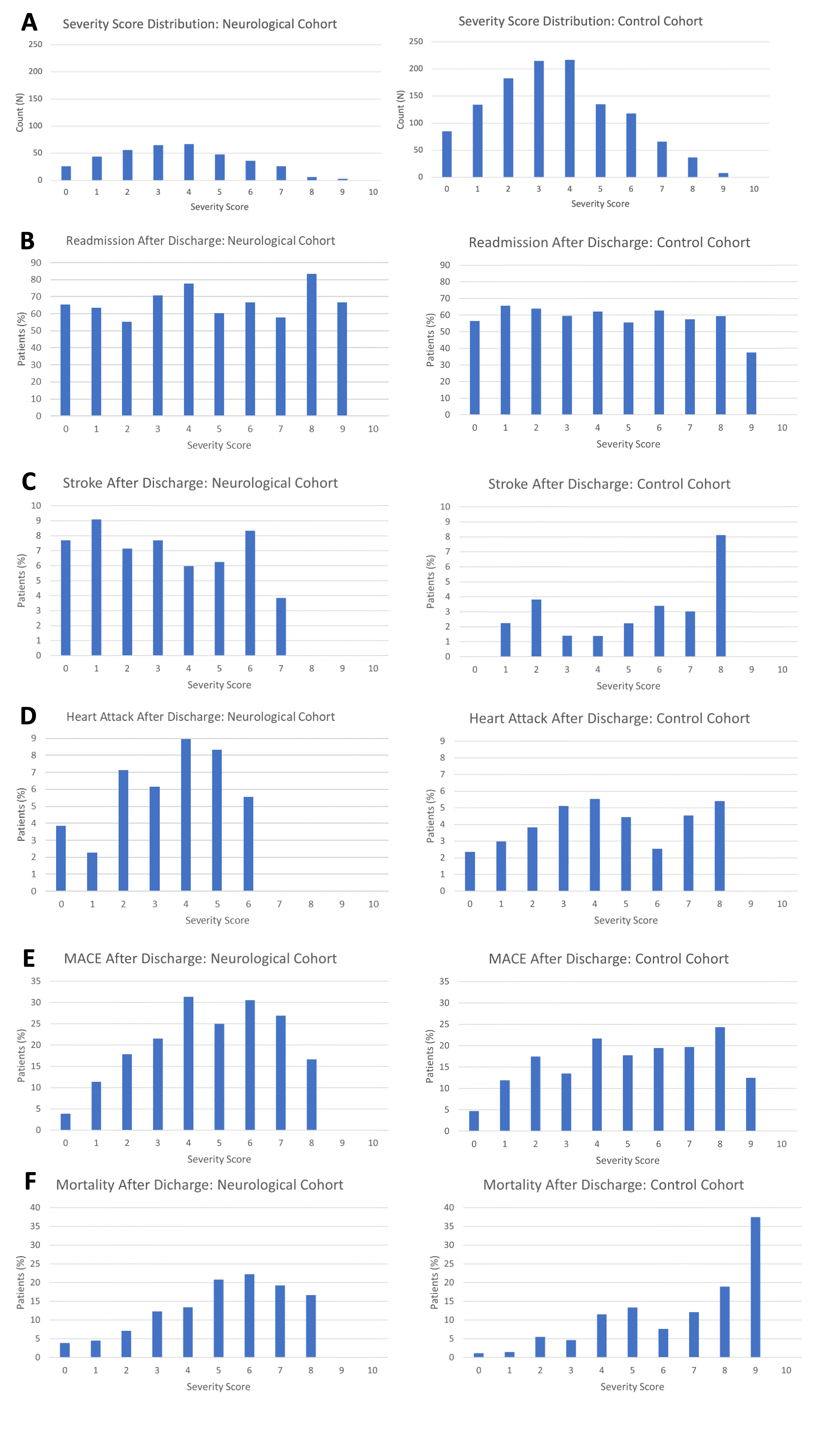

Supplement: S2 Fig — (A) Distribution of COVID-19 severity score in the neurological and control cohorts (survivors after COVID-19 hospitalization discharge). Percent of patients in the neurological and control cohorts who (B) were readmitted, (C) had stroke, (D) had heart attack, (E) had MACE, and (F) died after discharge from COVID-19 hospitalization. (TIFF) [file pmed.1004263.s006.tiff]
