# Supplementary material for: Long-term outcomes of hospitalized patients with SARS-CoV-2/COVID-19 with and without neurological involvement: 3-year follow-up assessment
Source: PLoS Med. 2024 Apr 4;21(4):e1004263. doi: 10.1371/journal.pmed.1004263 (PMC10994395; doi:10.1371/journal.pmed.1004263)
Supplement: Attachment — Submitted filename: Response Letter.docx [file pmed.1004263.s007.docx]

**Dear Editors and Reviewers,**

**We would like to thank you for your detailed and thoughtful comments. Please find the point-by-point responses and the revised manuscript. Only major changes are highlighted in the manuscript as there are numerous minor editorial changes.**

**Sincerely, Tim Duong on behalf of coauthors.**

      -----------------------------------------------------------
            Requests from the editors:

            GENERAL COMMENTS

            Please respond to all editor and reviewer comments.

            Please cite the reference numbers in square brackets (e.g., “We used the techniques developed by our colleagues [19] to analyze the data”). Citations should be preceding punctuation.

**Done as suggested.**

            Please cite your Supporting Information as outlined here: [https://nam04.safelinks.protection.outlook.com/?url=https%3A%2F%2Fjournals.plos.org%2Fplosmedicine%2Fs%2Fsupporting-information&data=05%7C01%7Canna.eligulashvili%40einsteinmed.edu%7C2b59ff936dcd4a60cfcf08dba8ac2661%7C9c01f0fd65e040c089a82dfd51e62025%7C0%7C0%7C638289229551308991%7CUnknown%7CTWFpbGZsb3d8eyJWIjoiMC4wLjAwMDAiLCJQIjoiV2luMzIiLCJBTiI6Ik1haWwiLCJXVCI6Mn0%3D%7C3000%7C%7C%7C&sdata=LiLQ9s2rI%2FD1hTLknYRSxhRHiyWXfrS25TG%2BkjQ2IpA%3D&reserved=0](https://nam04.safelinks.protection.outlook.com/?url=https%3A%2F%2Fjournals.plos.org%2Fplosmedicine%2Fs%2Fsupporting-information&data=05%7C01%7Ctim.duong%40einsteinmed.edu%7C2566d86c08f0446eec7608dba9b5f00a%7C9c01f0fd65e040c089a82dfd51e62025%7C0%7C0%7C638290371114971415%7CUnknown%7CTWFpbGZsb3d8eyJWIjoiMC4wLjAwMDAiLCJQIjoiV2luMzIiLCJBTiI6Ik1haWwiLCJXVCI6Mn0%3D%7C3000%7C%7C%7C&sdata=NSDWagl51GlGWGxAXkFmdjIcxj3%2FK%2BO0SoWQDiD0u%2Bg%3D&reserved=0)

**Done as suggested.**

            ACADEMIC EDITOR COMMENTS

            The manuscript could use more information on whether the participants were lost to follow up had different baseline characteristics, and why those who died during the hospitalization were excluded. The latter is relevant to the part regarding imaging, and how imaging findings or the presence of neurological injury led to withdrawal of care versus not withdrawing care, and those who did not have withdrawal could have been different from those of other centers who had imaging. Lastly there should be some context to this, any reason for admission is going to have worse outcomes in the long term when there is neurological dysfunction so that the findings may not be that surprising and it will be difficult to know if there is something unique about COVID with neurological injury as opposed to any other primary reason for admission.

            EDITORIAL COMMENTS

          E1  We feel that the manuscript could benefit from being more focused and from exploring and discussing the implications of the study results in more depth.

**Thank you for the incisive commentaries. We have appropriately revised the abstract, introduction and discussion. Major changes are highlighted within the revised manuscript.**

            EDITOR-IN-CHIEF COMMENTS

          EIC1 Please include reporting of acute and long-term neurological outcomes in your manuscript or discuss why this was not done in detail. In addition, please provide a detailed description of the propensity score matching and the optimisation of the matching used.

**This study focused on the relevant long-term outcomes. Acute outcomes of this cohort have been previously reported as referenced. However, we attempt to seamlessly weave a discussion of both short- and long-term findings and greater contextual details in the abstract, introduction and discussion. This has allowed us to define a new COVID-19 neurological syndrome distinct from ‘Long COVID’ with major implications for understanding disease pathogenesis and remediation and for giving rise to new areas for future research initiatives.**

**We employed the propensity matching utilized in our previous paper. Since our publication, there has not yet been external validation of our severity scoring system. We have elaborated on the propensity matching criteria below and within the revised manuscript.**

***COVID-19 disease severity score*: In-hospital COVID-19 disease severity scores [12] were the sum of five components that range from 0 to 10 points, on admission: (1) age by decile (age greater than 60, 70, and 80 years earned 1, 2, and 3 points, respectively), (2) mean arterial pressure (MAP) indicating hypotension (MAP below 80, 70, and 60 mmHg earned 1, 2, and 3 points, respectively), (3) oxygen saturation below 94% indicating impaired pulmonary function (1 point), (4) BUN greater than 30 mg/dL indicating impaired renal function (1 point), and (5) INR greater than 1.2 and CRP greater than 10 mg/L, indicating coagulopathy and inflammatory response. Scores ranged from 0 to 10 with higher score reflecting worse COVID-19 disease severity. For controls, propensity matching was performed based on this COVID-19 disease severity score. These scores have not been independently validated using external datasets.**

Thank you.

            DATA AVAILABILITY STATEMENT

            PLOS Medicine requires that the de-identified data underlying the specific results in a published article be made available, without restrictions on access, in a public repository or as Supporting Information at the time of article publication, provided it is legal and ethical to do so. Please see the policy at [https://nam04.safelinks.protection.outlook.com/?url=http%3A%2F%2Fjournals.plos.org%2Fplosmedicine%2Fs%2Fdata-availability&data=05%7C01%7Canna.eligulashvili%40einsteinmed.edu%7C2b59ff936dcd4a60cfcf08dba8ac2661%7C9c01f0fd65e040c089a82dfd51e62025%7C0%7C0%7C638289229551308991%7CUnknown%7CTWFpbGZsb3d8eyJWIjoiMC4wLjAwMDAiLCJQIjoiV2luMzIiLCJBTiI6Ik1haWwiLCJXVCI6Mn0%3D%7C3000%7C%7C%7C&sdata=qlC%2FgowKADhz8TJAnDWZ3ImvC1v0N0ZI9oTol0xL81Q%3D&reserved=0](https://nam04.safelinks.protection.outlook.com/?url=http%3A%2F%2Fjournals.plos.org%2Fplosmedicine%2Fs%2Fdata-availability&data=05%7C01%7Ctim.duong%40einsteinmed.edu%7C2566d86c08f0446eec7608dba9b5f00a%7C9c01f0fd65e040c089a82dfd51e62025%7C0%7C0%7C638290371114971415%7CUnknown%7CTWFpbGZsb3d8eyJWIjoiMC4wLjAwMDAiLCJQIjoiV2luMzIiLCJBTiI6Ik1haWwiLCJXVCI6Mn0%3D%7C3000%7C%7C%7C&sdata=3P8GOC83K6rRPOmN5bWUC57nOBCjmvgH%2FCNdOZ3WiX8%3D&reserved=0) and FAQs at [https://nam04.safelinks.protection.outlook.com/?url=http%3A%2F%2Fjournals.plos.org%2Fplosmedicine%2Fs%2Fdata-availability%23loc-faqs-for-data-policy&data=05%7C01%7Canna.eligulashvili%40einsteinmed.edu%7C2b59ff936dcd4a60cfcf08dba8ac2661%7C9c01f0fd65e040c089a82dfd51e62025%7C0%7C0%7C638289229551308991%7CUnknown%7CTWFpbGZsb3d8eyJWIjoiMC4wLjAwMDAiLCJQIjoiV2luMzIiLCJBTiI6Ik1haWwiLCJXVCI6Mn0%3D%7C3000%7C%7C%7C&sdata=A0lOO24IZ5fUbgl8xX4gWfXnciriJ13cy5D%2FkNZioBk%3D&reserved=0](https://nam04.safelinks.protection.outlook.com/?url=http%3A%2F%2Fjournals.plos.org%2Fplosmedicine%2Fs%2Fdata-availability%23loc-faqs-for-data-policy&data=05%7C01%7Ctim.duong%40einsteinmed.edu%7C2566d86c08f0446eec7608dba9b5f00a%7C9c01f0fd65e040c089a82dfd51e62025%7C0%7C0%7C638290371114971415%7CUnknown%7CTWFpbGZsb3d8eyJWIjoiMC4wLjAwMDAiLCJQIjoiV2luMzIiLCJBTiI6Ik1haWwiLCJXVCI6Mn0%3D%7C3000%7C%7C%7C&sdata=NopZUyBSFzMS2Lo5%2BVGkK5UyqS8i7Jv%2B%2FIdAWMNgfEQ%3D&reserved=0)

            The Data Availability Statement (DAS) requires revision. For each data source used in your study:

            a) If the data are freely or publicly available, note this and state the location of the data: within the paper, in Supporting Information files, or in a public repository (include the DOI or accession number).

**Done as suggested.**

            b) If the data are owned by a third party but freely available upon request, please note this and state the owner of the data set and contact information for data requests (web or email address). Note that a study author cannot be the contact person for the data.

**N/A**
            c) If the data are not freely available, please describe briefly the ethical, legal, or contractual restriction that prevents you from sharing it. Please also include an appropriate contact (web or email address) for inquiries (again, this cannot be a study author).

**N/A**
            For studies in which a novel model is central to the manuscript's findings, as is the case here, authors are responsible for providing the source code needed to replicate the study's findings in a repository (such as GitHub, SourceForge or Bitbucket) or a cloud computing service (such as Code Ocean). Protection of authors’ intellectual property will not be cause for exception. Please explain in the manuscript’s Data Availability Statement how readers can access the shared code.

            ABSTRACT

            Please structure your abstract using the PLOS Medicine headings (Background, Methods and Findings, Conclusions). The Methods and Findings sections should be combined into one section, “Methods and findings”.

**Done as suggested.**

            Please ensure that all numbers presented in the abstract are present and identical to numbers presented in the main manuscript text.

**Done as suggested.**

            PLOS Medicine requests that main results are quantified with 95% CIs as well as p values. When reporting p values please report as p<0.001 and where higher as the exact p value p=0.002, for example. Please provide specific P values in place of [NS or P>.05]. For the purposes of transparent data reporting, if not including the aforementioned please clearly state the reasons why not.

**Done as suggested throughout the manuscript.**

            Please include any important dependent variables that are adjusted for in the analyses.

**Done as suggested. Dependent variables were not adjusted for in any analyses due to the exploratory nature of the study.**

            Throughout, suggest reporting statistical information as follows to improve clarity for the reader “22% (95% CI [13%,28%]; p</=)”. Please amend throughout the abstract and main manuscript.

**Done as suggested.**

            Please note the use of commas to separate upper and lower bounds, as opposed to hyphens as these can be confused with reporting of negative values.

**Done as suggested.**

            Please define all abbreviations used for statistical reporting at first use (HR, CI, OR).

**Done as suggested.**

            When a p value is given, please specify the statistical test used to determine it.

**Done as suggested.**

            Please include the study design and study population, i.e. please provide brief demographic details of the study population (e.g. sex, age, ethnicity, etc).

**Done as suggested.**

            In the last sentence of the Abstract Methods and Findings section, please describe the main limitation(s) of the study's methodology.

**Done as suggested.**

            l.71: Please define ‘vs’ at first use.

**Done as suggested.**

            l.86: Please change ‘have’ to ‘had’.

**Done as suggested.**

            AUTHOR SUMMARY

            At this stage, we ask that you include a short, non-technical Author Summary of your research to make findings accessible to a wide audience that includes both scientists and non-scientists. The Author Summary should immediately follow the Abstract in your revised manuscript. This text is subject to editorial change and should be distinct from the scientific abstract. Please see our author guidelines for more information: [https://nam04.safelinks.protection.outlook.com/?url=https%3A%2F%2Fjournals.plos.org%2Fplosmedicine%2Fs%2Frevising-your-manuscript%23loc-author-summary&data=05%7C01%7Canna.eligulashvili%40einsteinmed.edu%7C2b59ff936dcd4a60cfcf08dba8ac2661%7C9c01f0fd65e040c089a82dfd51e62025%7C0%7C0%7C638289229551308991%7CUnknown%7CTWFpbGZsb3d8eyJWIjoiMC4wLjAwMDAiLCJQIjoiV2luMzIiLCJBTiI6Ik1haWwiLCJXVCI6Mn0%3D%7C3000%7C%7C%7C&sdata=uPJsqscYryGfJTqrgkH%2FFZV%2FDHME7KnwexTZyqfj1TI%3D&reserved=0](https://nam04.safelinks.protection.outlook.com/?url=https%3A%2F%2Fjournals.plos.org%2Fplosmedicine%2Fs%2Frevising-your-manuscript%23loc-author-summary&data=05%7C01%7Ctim.duong%40einsteinmed.edu%7C2566d86c08f0446eec7608dba9b5f00a%7C9c01f0fd65e040c089a82dfd51e62025%7C0%7C0%7C638290371114971415%7CUnknown%7CTWFpbGZsb3d8eyJWIjoiMC4wLjAwMDAiLCJQIjoiV2luMzIiLCJBTiI6Ik1haWwiLCJXVCI6Mn0%3D%7C3000%7C%7C%7C&sdata=E9entaArVD656PY39wbiYi44l9H0f%2Fv8JyudCdKtE9c%3D&reserved=0).

            The summary should include 2-3 single sentence, individual bullet points under each of the questions.
            It may be helpful to review currently published articles for examples which can be found on our website here [https://nam04.safelinks.protection.outlook.com/?url=https%3A%2F%2Fjournals.plos.org%2Fplosmedicine%2F&data=05%7C01%7Canna.eligulashvili%40einsteinmed.edu%7C2b59ff936dcd4a60cfcf08dba8ac2661%7C9c01f0fd65e040c089a82dfd51e62025%7C0%7C0%7C638289229551308991%7CUnknown%7CTWFpbGZsb3d8eyJWIjoiMC4wLjAwMDAiLCJQIjoiV2luMzIiLCJBTiI6Ik1haWwiLCJXVCI6Mn0%3D%7C3000%7C%7C%7C&sdata=hgbeSON8zHdR9avjRA6u7qL4Sb0XrW5eJApIRIAdlM8%3D&reserved=0](https://nam04.safelinks.protection.outlook.com/?url=https%3A%2F%2Fjournals.plos.org%2Fplosmedicine%2F&data=05%7C01%7Ctim.duong%40einsteinmed.edu%7C2566d86c08f0446eec7608dba9b5f00a%7C9c01f0fd65e040c089a82dfd51e62025%7C0%7C0%7C638290371114971415%7CUnknown%7CTWFpbGZsb3d8eyJWIjoiMC4wLjAwMDAiLCJQIjoiV2luMzIiLCJBTiI6Ik1haWwiLCJXVCI6Mn0%3D%7C3000%7C%7C%7C&sdata=WewrUum9Dt9hHRDGGN5wyB4BCeG%2BYb0A5FOF%2FiaX2DE%3D&reserved=0)

**Done as suggested.**

            INTRODUCTION

            Please address past research and explain the need for and potential importance of your study. Indicate whether your study is novel and how you determined that. If there has been a systematic review of the evidence related to your study (or you have conducted one), please refer to and reference that review and indicate whether it supports the need for your study.

            Please conclude the Introduction with a clear description of the study question or hypothesis.

**The following is added:**

“The goal of this study was to evaluate the 3-year outcomes of COVID-19 patients with significant neurological complaints that warranted neuroimaging during COVID-19 when compared with propensity-matched controls without significant neurological complaints. Improved understanding of the long-term outcomes of COVID-19 patients with CNS manifestations could help to identify at-risk patients and enable timely interventions to address the potentially high burden of care among these COVID-19 survivors. We hypothesized that COVID-19 patients with significant neurological complaints have worse outcomes up to 3-years follow-up.”

            METHODS AND RESULTS

            Please ensure that the study is reported according to the STROBE guideline, and include the completed STROBE checklist as Supporting Information.  Please add the following statement, or similar, to the Methods: “This study is reported as per the Strengthening the Reporting of Observational Studies in Epidemiology (STROBE) guideline (S1 Checklist).“
            The STROBE guideline can be found here: [https://nam04.safelinks.protection.outlook.com/?url=http%3A%2F%2Fwww.equator-network.org%2Freporting-guidelines%2Fstrobe%2F&data=05%7C01%7Canna.eligulashvili%40einsteinmed.edu%7C2b59ff936dcd4a60cfcf08dba8ac2661%7C9c01f0fd65e040c089a82dfd51e62025%7C0%7C0%7C638289229551308991%7CUnknown%7CTWFpbGZsb3d8eyJWIjoiMC4wLjAwMDAiLCJQIjoiV2luMzIiLCJBTiI6Ik1haWwiLCJXVCI6Mn0%3D%7C3000%7C%7C%7C&sdata=pbbjkaoIrNbAGfMGFaj2airNLRiZBQfZKUVM0P1KOOM%3D&reserved=0](https://nam04.safelinks.protection.outlook.com/?url=http%3A%2F%2Fwww.equator-network.org%2Freporting-guidelines%2Fstrobe%2F&data=05%7C01%7Ctim.duong%40einsteinmed.edu%7C2566d86c08f0446eec7608dba9b5f00a%7C9c01f0fd65e040c089a82dfd51e62025%7C0%7C0%7C638290371114971415%7CUnknown%7CTWFpbGZsb3d8eyJWIjoiMC4wLjAwMDAiLCJQIjoiV2luMzIiLCJBTiI6Ik1haWwiLCJXVCI6Mn0%3D%7C3000%7C%7C%7C&sdata=44rId1%2BBA5j5pSGqunALNT%2Bi%2FEvdttJSP4zAwBcq1Kc%3D&reserved=0)
            When completing the checklist, please use section and paragraph numbers, rather than page numbers.

**STROBE is included.**

            Did your study have a prospective protocol or analysis plan? Please state this (either way) early in the Methods section.

**Not applicable.**

            a) If a prospective analysis plan (from your funding proposal, IRB or other ethics committee submission, study protocol, or other planning document written before analyzing the data) was used in designing the study, please include the relevant prospectively written document with your revised manuscript as a Supporting Information file to be published alongside your study, and cite it in the Methods section. A legend for this file should be included at the end of your manuscript.

**Not applicable.**

            b) If no such document exists, please make sure that the Methods section transparently describes when analyses were planned, and when/why any data-driven changes to analyses took place.

**Not applicable.**

            c) In either case, changes in the analysis-- including those made in response to peer review comments-- should be identified as such in the Methods section of the paper, with rationale.

**Not applicable.**

            For all observational studies, in the manuscript text, please indicate: (1) the specific hypotheses you intended to test, (2) the analytical methods by which you planned to test them, (3) the analyses you actually performed, and (4) when reported analyses differ from those that were planned, transparent explanations for differences that affect the reliability of the study's results. If a reported analysis was performed based on an interesting but unanticipated pattern in the data, please be clear that the analysis was data-driven.

**Done as suggested. The specific hypotheses we intended to test are in the concluding sentence of the introduction. The analytical methods and analyses are detailed in the *Primary Outcomes* and *Statistical Analysis* paragraphs in the Methods. Reported analyses did not differ from those that were planned because the analysis was data-driven.**

            PLOS Medicine requests that main results are quantified with 95% CIs as well as p values.  When reporting p values please report as p<0.001 and where higher as the exact p value p=0.002, for example.  For the purposes of transparent data reporting, if not including the aforementioned please clearly state the reasons why not.

**Done as suggested.**

            Please include any important dependent variables that are adjusted for in the analyses.

**No dependent variables were adjusted for in the analyses.**

            Suggest reporting statistical information as detailed above – see under ABSTRACT

**Done as suggested.**

            Please define "lost to follow-up" as used in this study. Other reasons for exclusion should be defined.

**These patients did not return to our health system. Done as suggested.**

            Please define the length of follow up (eg, in mean, SD, and range).

**Done as suggested.** **The length of follow-up was defined as the time elapsed between patient COVID-19 hospitalization discharge and either date of death or date of most recent patient encounter up to January 23, 2023. The average length of follow-up was 602±400 and 672±269 days for the neurological and control cohorts, respectively. The average range of follow-up was (0.138, 1037.413 days) and (0.063, 1043.25 days) for the neurological and control cohorts, respectively.**

            Please provide the actual numbers of events for the outcomes, not just summary statistics or ORs.

**The actual numbers of events for the outcomes for all variables can be found in the Tables.**

            Please present numerators and denominators for percentages, at least in the Tables [not
            necessarily each time they're mentioned].

**The numerators and denominators for percentages are found in the tables. The numerator is in the row corresponding to the variable of interest, and the denominator is located in the column header.**

            l.115: Please define ‘PCR’.

**Done as suggested.**

            l.161: Please insert a comma following “lymphocyte count (Lymph)”.

**Done as suggested.**

            ll.166-167: When describing age, please ensure to add a unit, such as ‘years’. Please revise throughout the entire manuscript.

**Done as suggested.**

            l.168: Please add a unit for mean arterial pressure (MAP).

**Done as suggested.**

            l.169: Please add a unit for blood urea nitrogen (BUN).

**Done as suggested.**

            l.170: Please add a unit for C-reactive protein (CRP).

**Done as suggested.**

            ll.181-182 please change to: “Major findings on both CT and MRI, were documented for their absence or presence of hemorrhage, active stroke, prior stroke, mass effect, and microhemorrhage.”

**Done as suggested.**

            l.186: Please clarify that you refer to COVID-19 hospitalization. Please revise throughout the entire manuscript.

**Done as suggested.**

            l.195: The statement “hospital readmission post discharge January 23, 2023 (3 years post COVID-19).” might be misleading and we suggest changing it to “hospital readmission post discharge until January 23, 2023 (3 years post COVID-19).”.

**Done as suggested.**

            l.202: Please change “confidential interval” to “confidence interval”.

**Done as suggested.**

            Please provide the name(s) of the institutional review board(s) that provided ethical approval.

**Done as suggested.**

            ll.222-223: Please remove the “Data Availability Statement” from the main manuscript. This information should only be included in the corresponding section in the online submission form.

**Done as suggested.**

            l.227/228: Please change ‘our’ to ‘the’.

**Done as suggested.**

            l.239: Please add a unit for age.

**Done as suggested.**

            l.240: Please exchange ‘gender’ with ‘sex’.

**Done as suggested.**

            ll.252-254 suggest: “Mortality rates post COVID-19 hospitalization were also higher in the neurological cohort compared to the control cohort at 0.5 years (7.73% vs 3.67%, p=0.0004), 1 year (9.18% vs 4.92%, p=0.0009), and 3 years (14.01% vs 7.84%, p=0.0001) follow-up.” or similar.

**Done as suggested.**

            l.262: Please change the reference to ‘Supplementary Figure 1B-F’.

**Done as suggested.**

            l.278: Please change ‘compared’ to ‘compares’.

**Done as suggested.**

            l.279: Please change ‘are’ to ‘were’.

**Done as suggested.**

            ll.278-284: When possible, please report the quantified main results with 95% CIs as well as p values. Please revise throughout the entire manuscript.

**Done as suggested. 95% CIs are located in Tables 1 and 2 (main results).**

            ll.288-291 suggest: “These variables included belonging the neurological cohort (OR=1.802 (1.237, 2.608), p=0.002), discharge disposition (OR=1.508, 95% CI=(1.276, 1.775), p<0.0001), congestive heart failure (OR=2.281 (1.429, 3.593), p=0.0004), COVID-19 severity score (OR=1.177 (1.062, 1.304), p=0.002), and age (OR=1.027 (1.010, 1.044), p=0.002).”

**Done as suggested.**

            ll.291-292: The term "trend" is used to refer to a nonsignificant P value. The term trend should be used only when the test for trend has been conducted. Please revise accordingly.

**The wording was removed.**

            l.305: Please add ‘control’ to “[…] score of 0: 42.36% neurological vs. 53.78%, p=0.037).”.

**Done as suggested.**

            ll.295-314: Please ensure that you are clear when describing the groups you are referring to, as you may vary the comparison throughout the manuscript, e.g. 'survivors versus non-survivors' and 'neurological versus control cohort'.

**Done as suggested.**

            ll.308-314: Please be sure to include details of the statistical results (and not only for the first bracket), i.e. the groups to which the results presented belong (neurological, control, etc.).

**Done as suggested.**

            DISCUSSION

            Please present and organize the Discussion as follows: a short, clear summary of the article's findings; what the study adds to existing research and where and why the results may differ from previous research; strengths and limitations of the study; implications and next steps for research, clinical practice, and/or public policy; one-paragraph conclusion.

**Done as suggested.**

            Please remove any subheadings.

**Done as suggested.**

            l.337: Please change ‘our’ to ‘the’.

**Done as suggested.**

            l.348: In line 338 you refer to the whole group of participants (neurological and control cohorts) as one cohort (our cohort), whereas here you refer to the whole group as 'both cohorts'. Please revise to ensure clarity (throughout the entire manuscript).

**Done as suggested.**

            l.359: Please add a reference for “This is widely seen in other medical conditions such as urosepsis.”.

**This statement is deleted.**

            l.361/363: Please exchange ‘gender’ with ‘sex’. Please revise throughout the entire manuscript.

**Done as suggested.**

            ll.367-368: Please change to: “Belonging to the neurological cohort was also an independent risk factor for post-discharge mortality.”

**Done as suggested.**

            l.372: Please ensure that you use a consistent spelling when writing 'post discharge' ('post discharge' or 'post-discharge'). Please revise throughout the entire manuscript.

**Done as suggested.**

            ll.388-390: Please change to “However, there were generally no group differences in either qualitative or score-based findings, except that the neurological cohort showed greater volume loss post-COVID-19 compared to controls.”.

**Done as suggested.**

            l.397: Please temper claims of primacy of results by stating, "to our knowledge" or something similar.

**Done as suggested.**

            ll.413-420: Please add according references.

**Done as suggested.**

            ll.442-444 suggest: “The mixture of MRI and CT may have different sensitivities for various accompanying pathologies and more sophisticated imaging techniques may be warranted.”

**Done as suggested.**

            Please remove the ‘Study funding’ section. This information should only be included in the corresponding section in the online submission form.

**Done as suggested.**

            TABLES

            Please provide titles and descriptions for all tables (including those in Supporting Information files).

**Done as suggested.**

            Please define abbreviations used in the table of each table (including those in Supporting Information files).

**Done as suggested.**

            When reporting p-values in tables (including those in Supporting Information files), please include a separate column (in each table) with the p-values and report as <0.001 or the exact p-value where higher and remove all reference to asterisks and p-value thresholds.

**Done as suggested.**

            Table 1: Please define ‘SD’, ‘COPD’, ‘CHF’, CKD’, ‘SNF’, ‘MACE’.

**Done as suggested.**

            Table 1: For consistency, please change the column header to ‘Neurological Cohort’.

**Done as suggested.**

            Table 2: Please define ‘ARDS’, ‘GI’, ‘ICH’

**Done as suggested.**

            Table 3: Please change ‘year old’ to ‘years old’.

**Done as suggested.**

            Table 3: Please define ‘SD’, ‘COPD’, ‘SNF’, ‘OR’, ‘CI’, ‘p-val’.

**Done as suggested.**

            Table 3: In (B), please add ‘Sex’ to ‘Male’.

**Done as suggested.**

            Table 4: Please note that the main text refers to 'Table 4A' and 'Table 4B', whereas the actual table is divided into 'Table 4' and 'Table 4B'.

**Done as suggested.**

            Table 4: In the title you refer to ‘COVID-19 infection’, whereas in the manuscript you refer to ‘COVID-19 hospitalization’. Please revise accordingly.

**Done as suggested.**

            Table 4: For clarity, please describe ‘Studies’ as ‘Imaging studies’ or similar.

**Done as suggested.**

            Table 4: Please define ‘MRI’, ‘CT’, ‘p-val’. We suggesting writing ‘p-value’ in full in the column header.

**Done as suggested.**

            Table 4: Please define the meaning of the numbers following ‘±’ (e.g. standard deviation).

**Done as suggested.**

            Table 4B: For clarity, please describe ‘Studies’ as ‘Imaging studies’ or similar.

**Done as suggested.**

            Table 4B: In the table description or the column headers, please ensure to mention that you are referring to the different cohorts.

**Done as suggested.**

            Table 4B: Please define the meaning of the numbers following ‘±’ (e.g. standard deviation).

**Done as suggested.**

            Supplemental Table 1: Please ensure to define all abbreviations (including units) used in the table.

**Done as suggested.**

            Supplemental Table 1: For consistency, please change the column header to ‘Neurological Cohort’.

**Done as suggested.**

            FIGURES

            For all Figures, please ensure that you have complied with our figures requirements [https://nam04.safelinks.protection.outlook.com/?url=http%3A%2F%2Fjournals.plos.org%2Fplosmedicine%2Fs%2Ffigures&data=05%7C01%7Canna.eligulashvili%40einsteinmed.edu%7C2b59ff936dcd4a60cfcf08dba8ac2661%7C9c01f0fd65e040c089a82dfd51e62025%7C0%7C0%7C638289229551308991%7CUnknown%7CTWFpbGZsb3d8eyJWIjoiMC4wLjAwMDAiLCJQIjoiV2luMzIiLCJBTiI6Ik1haWwiLCJXVCI6Mn0%3D%7C3000%7C%7C%7C&sdata=uvg1qANLvR8xyWPLqwurlyMFZ5hOnH85HxDT55h1Fbk%3D&reserved=0](https://nam04.safelinks.protection.outlook.com/?url=http%3A%2F%2Fjournals.plos.org%2Fplosmedicine%2Fs%2Ffigures&data=05%7C01%7Ctim.duong%40einsteinmed.edu%7C2566d86c08f0446eec7608dba9b5f00a%7C9c01f0fd65e040c089a82dfd51e62025%7C0%7C0%7C638290371114971415%7CUnknown%7CTWFpbGZsb3d8eyJWIjoiMC4wLjAwMDAiLCJQIjoiV2luMzIiLCJBTiI6Ik1haWwiLCJXVCI6Mn0%3D%7C3000%7C%7C%7C&sdata=QMFwboqpcuHxDRi%2BCFqRv%2BT4qPFFrsluObrY%2FlnRFDE%3D&reserved=0).

**Done as suggested.**

            Please provide titles and legends for all figures (including those in Supporting Information files).

**Done as suggested.**

            Please define abbreviations used in the table/figure legend of each figure and/or table (including those in Supporting Information files).

**Done as suggested.**

            Please consider avoiding the use of red and green in order to make your figure more accessible to those with colour blindness.

**Done as suggested.**

            Figure 2: In the Kaplan-Meier curve, please provide the number at risk for each time interval.

**Done as suggested in Figure 2.**

            Figure 2: Please indicate in the figure caption the meaning of the whiskers.

**Done as suggested.**

            Figure 2: Please show the axis beginning at zero. If this is not possible, please show a break in the axis.

**Done as suggested.**

            Supplemental Figure 1: Please increase the size of the graphs.

**Done as suggested.**

            Supplemental Figure 1: We suggest changing the axis title to ‘Patient Count (N)’.

**It may be easier to compare differences between neurological and control cohorts if the axes are kept in Patient Count (%) because the two groups have different denominators.**

            REFERENCES

            Please ensure that journal name abbreviations match those found in the National Center for Biotechnology Information (NCBI) databases (<https://nam04.safelinks.protection.outlook.com/?url=http%3A%2F%2Fwww.ncbi.nlm.nih.gov%2Fnlmcatalog%2Fjournals&data=05%7C01%7Canna.eligulashvili%40einsteinmed.edu%7C2b59ff936dcd4a60cfcf08dba8ac2661%7C9c01f0fd65e040c089a82dfd51e62025%7C0%7C0%7C638289229551308991%7CUnknown%7CTWFpbGZsb3d8eyJWIjoiMC4wLjAwMDAiLCJQIjoiV2luMzIiLCJBTiI6Ik1haWwiLCJXVCI6Mn0%3D%7C3000%7C%7C%7C&sdata=TSVAP%2BCkjECGtmnQeVk%2FkbM0yx0XwBUznBfuDX11g9Q%3D&reserved=0>) and are appropriately formatted and capitalised.

            Please use the "Vancouver" style for reference formatting, and see our website for other reference guidelines [https://nam04.safelinks.protection.outlook.com/?url=https%3A%2F%2Fjournals.plos.org%2Fplosmedicine%2Fs%2Fsubmission-guidelines%23loc-references&data=05%7C01%7Canna.eligulashvili%40einsteinmed.edu%7C2b59ff936dcd4a60cfcf08dba8ac2661%7C9c01f0fd65e040c089a82dfd51e62025%7C0%7C0%7C638289229551308991%7CUnknown%7CTWFpbGZsb3d8eyJWIjoiMC4wLjAwMDAiLCJQIjoiV2luMzIiLCJBTiI6Ik1haWwiLCJXVCI6Mn0%3D%7C3000%7C%7C%7C&sdata=xNnJ8%2B%2B9D0ffQJjjUP5bWFE17qJyeNi%2F8wSlsBe8OqM%3D&reserved=0](https://nam04.safelinks.protection.outlook.com/?url=https%3A%2F%2Fjournals.plos.org%2Fplosmedicine%2Fs%2Fsubmission-guidelines%23loc-references&data=05%7C01%7Ctim.duong%40einsteinmed.edu%7C2566d86c08f0446eec7608dba9b5f00a%7C9c01f0fd65e040c089a82dfd51e62025%7C0%7C0%7C638290371114971415%7CUnknown%7CTWFpbGZsb3d8eyJWIjoiMC4wLjAwMDAiLCJQIjoiV2luMzIiLCJBTiI6Ik1haWwiLCJXVCI6Mn0%3D%7C3000%7C%7C%7C&sdata=Wc4OBHFTkYSqy37vBkcqJoDRd1pT%2B0vGFEa0lTLl438%3D&reserved=0)

**Done as suggested.**

            Where website addresses are cited, please specify the date of access.

**Not applicable.**

            Comments from the reviewers:

            Reviewer #1: The present study investigated the long-term consequences of patients with and without significant neurological manifestations during the initial hospitalization for COVID-19 over a period of three years. Acute neurological manifestations are a common complication of acute COVID-19 disease. The results indicate that COVID-19 patients with neurological manifestations have worse long-term outcomes compared to matched control groups. These findings suggest the importance of close monitoring and timely interventions for COVID-19 patients with neurological manifestations. Raising awareness and implementing appropriate measures are crucial to minimize the long-term effects.

            However, some important questions are not addressed or discussed in the study. Specifically, information about additional vaccinations over a three-year period is crucial and could have a significant impact on the longterm results. It would be worthwhile to at least discuss and consider how such vaccinations might affect the disease trajectory.

**We agree. We add such a discussion in the limitation section.**

Other factors such as reinfection, COVID-19 vaccination status, and influenza vaccination status could affect long-term outcomes. Most COVID-19 patients were early in the pandemic before vaccines became available. It is likely COVID-19 vaccines and improved COVID-19 treatments will likely reduce long-term neurological sequela.

            The gender-specific differences in the results are indeed very interesting and should be further investigated. A detailed analysis of the current literature on this topic could provide valuable insights and contribute to a better understanding of these differences.

**We agree that sex differences in COVID-19 outcomes are very interesting and have been reported in the literature. In our cohorts, we found no significant sex differences in composition and outcomes.**

            Furthermore, it is important to mention that the observation period was limited to the early phase of the pandemic, focusing on the Wuhan variant, which was considered more aggressive compared to later virus variants. This fact should be taken into account in the discussion and the potential implications of this specific virus variant on the results of the manuscript should be discussed.

**We agree. The following has been added.**

Other factors such as reinfection, COVID-19 vaccination status, and influenza vaccination status could affect long-term outcomes. Most COVID-19 patients were early in the pandemic before vaccines became available. It is likely COVID-19 vaccines and improved COVID-19 treatments will likely reduce long-term neurological sequela.

            Overall, this is an interesting study with a large number of patients and a long observation period. However, the aforementioned points are important aspects that should be considered in a comprehensive discussion to further improve the understanding of the results. In the abstract, it should be mentioned which neurological disorders were investigated during the acute phase.

**Thank you.** These neurological disorders were investigated up to Jan 23, 2023. **Done as suggested.**

            In Table 2, it should be clarified within which timeframe the causes of death after discharge were recorded. Did they encompass the entire three-year observation period or were they deaths that occurred immediately after discharge?

**These primary causes of death of patients represented those who died after discharge up to January 23, 2023.**

            In line 240 on page 10, the gender composition should be specified more precisely to avoid misunderstandings.

**Done as suggested.**

Reviewer #2: Statistical review

            This paper reports a retrospective cohort study assessing difference in post-discharge outcomes amongst individuals hospitalised for covid-19 with and without neurological symptoms. I had some comments on the statistical methods and reporting in the paper, which are listed below.

            1.      Abstract/results: For comparative results it would be useful to provide a between group difference measure (e.g odds ratio) and 95% CI as well as p-value.

**The 95% CIs are provided.**

            2.      Abstract methods: can a bit more be provided about the methods used to make inference? 'predictive models' is a big vague.

**We have revised the manuscript to state it is logistic regression.**

            3.      Abstract line 77 - could it be made clear that the percentages of mortality due to the listed reasons are across the two cohorts e.g. 'Across both cohorts, the major causes of death…'

**Done as suggested.**

            4.      Abstract line 79: were these results from univariable models or multivariable models?

**Done as suggested. The results are from univariate logistic regression models.**

            5.      Abstract line 84: if a p-value is to be reported in the abstract, can the brain volume loss be reported similarly to other variables (i.e. difference in groups and more exact p-value).

**Done as suggested.**

            6.      Page 6: could it be clarified that the controls were hospitalised with covid over the same time period as the neurological cohort? If not, this would be somewhat of a limitation of the comparison.

**Yes over the time period. Thank you.**

            7.      Page 9: when were the listed secondary outcomes measured?

**Done as suggested. Secondary outcomes were measured after COVID-19 hospitalization discharge up to January 23, 2023 (3 years post COVID-19).**

            8.      Page 9, line 199: I wasn't sure why this paragraph was included here rather than in 'Statistical analysis'?

**This paragraph about the scoring methods is more related to the protocol of scoring than the statistical analysis of the scores.**

            9.      Page 9: It wasn't clear to me which variables were adjusted for in the logistic and Cox models.

**Done as suggested. In the logistic regression model, death after COVID-19 hospitalization discharge was the dependent variable and flag. Independent variables included belonging to the neurological cohort, discharge disposition, congestive heart failure, COVID-19 severity score, age, male sex, chronic kidney disease, diabetes, COPD/asthma, and hypertension.**

            10.     Page 10 line 226: I wasn't sure how to interpret the numbers here. Does it indicate that the number 'returned to our health system' was the number in subsequent analyses, with 10.6% attrition representing the proportion discharged alive who weren't included in the analysis? It might be useful to add to the statistical analysis section which individuals were included in the analysis and how attrition was dealt with for the different types of outcomes.

**Done as suggested. We defined the attrition rate as the ratio of patients who did not return to the health system (43 neurological, 156 control) divided by the number of patients discharged alive (414 neurological, 1199 control).**

            11.     Page 10 line 239: I don't think adding in 'p>0.05' for the results here is necessary. Perhaps including the p-values in Table 1 would be better?

**Done as suggested.**

            12.     Table 2 - I would recommend reporting estimated differences between arms, 95% CIs and p-values in this table.

**95% CIs and p-values reported.**

            13.     Line 255 "cohort had a significantly lower survival probability than the control cohort at all timepoints" - I would change this to 'cohort had a significantly lower time to death than the control cohort'

**Done as suggested.**

            14.     Table 3B - are these results from a multivariable logistic regression? If so I would urge the authors to bear in mind the table 2 fallacy which would suggest that one should not overinterpret the individual variable odds ratios. If the main interest is in the neurological vs control comparison on mortality, then the model should be careful not to adjust for mediators (perhaps discharge disposition and congestive heart failure, unless that was from prior to hospitalisation?) otherwise the results are prone to bias from the table 2 fallacy. If the results are similar with not adjusting for these then this would be good to note. I would also recommend that the abstract and discussion addresses this issue (in particular the 'discharge to skilled nursing facilities').

**The results in Table 3B are coming from a univariate logistic regression where the outcome dependent variable was death after COVID-19 hospitalization discharge.**

            15.     Page 13: For the imaging analysis, it seems a high proportion of controls did not have results. As a result, these results seem less reliable to me - I would recommend caveating these results or mentioning assumptions made in the analysis.

**Thank you. This is added.**

Another limitation is that many controls did not have imaging results and thus findings need to be interpreted with caution.

            James Wason


            Reviewer #3: I appreciate the opportunity to review the manuscript titled "Long-term outcomes of hospitalized SARS-CoV-2/COVID-19 patients with and without neurological involvement: 3-year follow-up assessment" by Eligulashvili. et. al. In this matched cohort study, the authors assessed long-term outcomes of patients admitted with COVID-19 based on presence or absence of neurological symptoms. The authors found patient with neurological symptoms where less likely to be discharge home, readmitted more, had higher incidence of mortality and stroke.
            I commend the authors for performing an extensive analysis of the dataset which significantly add to our current understanding of the deleterious effects of development of neurological symptoms in COVID-19, however they remain some major issues that needs to be resolved.

            Major concerns
            1.      Propensity score matching was based on age and covid-19 severity score.
            The reference is for the previous article published by the group. Can you please reference the validation of this severity scoring system.
            Also, it will be beneficial to know how well the propensity score matched the patient population.

            Another table in the supplement with information on the matched group will be highly helpful

**Since our publication, there has not yet been external validation of our severity scoring system.**

**There was 1:1 exact match as defined in the original paper. We did not change the propensity match criteria.**

**After matching, all demographic variables were not significantly different between groups.**

| Age, years old (at admission) | 69.71±15.80 | 70.26±15.14 | 0.539 |
| --- | --- | --- | --- |
| Female sex (%) | 186 (44.93%) | 578 (48.21%) | 0.249 |
| Combined Race and Ethnicity |  |  |  |
| White, not Hispanic | 27 (6.52%) | 94 (7.84%) | 0.380 |
| Black, not Hispanic | 176 (42.51%) | 432 (36.03%) | **0.019** |
| Hispanic | 139 (33.57%) | 458 (38.20%) | 0.093 |
| Other | 62 (14.98%) | 169 (14.10%) | 0.659 |
| **Comorbidities** (at admission) | | |  |
| Hypertension | 186 (44.93%) | 523 (43.62%) | 0.644 |
| COPD/Asthma | 42 (10.14%) | 144 (12.01%) | 0.306 |
| Diabetes | 126 (30.43%) | 343 (28.61%) | 0.480 |
| CHF | 49 (11.84%) | 118 (9.84%) | 0.251 |
| CKD | 90 (21.74%) | 223 (18.60%) | 0.164 |

            2.      A single bucket of neurological symptoms which range from stroke, seizure to impaired arousal and neuro-COVID-19 complex may limit which of these neurological symptoms are truly associated with worse outcome. A subgroup analysis based on structural vs non-structural pathology may be helpful. As it is difficult to ascertain that having delirium or vertigo during the stay is associated same long-term as having seizures, stroke or brain tumor.

**We agree with your comments. Moreover, we do not have statistical power to attribute outcomes to different neurological pathology, which were highly heterogeneous, although the initial study did find a subset of neurological syndromes associated with more adverse outcomes. This suggests that specific forms of COVID-19-mediated brain pathology are preferentially associated with critical care illness and death through mechanisms of neural-systemic crosstalk that are not captured by traditional measures of critical care illness as determined by our severity score index. More detailed assessment of these potentially novel mechanisms will require further large and prospective investigations.**

            3.      Flow diagram on the initial and final sample size will be very helpful.

**Done as suggested. Included as Supplemental Figure 1.**

            4.      One of the most common causes of admission in COVID-19 was respiratory failure, however the data on their respiratory status was lacking. Providing and controlling for respiratory factor particularly need for mechanical ventilation, duration of mechanical ventilation, need for tracheostomy, major life sustaining support including dialysis, ECMO are needed to truly needed to better understand if the effect on discharge, readmission, stroke and mortality being seen is completely driving by neurological symptoms or other confounders.

**We agree with your comments. However, future examination of these granular data would require larger sample sizes and prospective study design. Moreover, we do not have statistical power to attribute outcomes to different respiratory factors. Thus, we can only account for via the COVID-19 severity score.**

            5.      The article title suggests long-term effect, however looking at the Kaplan-Meier survival analysis, a significant number of death are happening within 100 days. Most studies have used after 90 days or 6 months from discharge to determine long-term mortality (PMID: 21247589,  PMID: 33643222). Please change the title and also, if the authors can provide more based on time period of 90 days post discharge to 1 yr, 2 yr and 3 yr, the information will be very helpful to the readers.

**Thank you for the references. They are cited.**

**Durations post-covid reported in the literature have varied since the pandemic is still evolving.**

**Because our study investigates the primary and secondary outcomes up to 3 years post-discharge, we feel the title of long-term effects is appropriate.**
