# Supplementary material for: Long-term outcomes of hospitalized patients with SARS-CoV-2/COVID-19 with and without neurological involvement: 3-year follow-up assessment
Source: PLoS Med. 2024 Apr 4;21(4):e1004263. doi: 10.1371/journal.pmed.1004263 (PMC10994395; doi:10.1371/journal.pmed.1004263)
Supplement: Attachment — Submitted filename: Response Letter.docx [file pmed.1004263.s008.docx]

Requests from the editors:

    MAIN POINTS

    1) The Editors agree with Reviewer 3 with regard to the reporting of respiratory variables. The initial review stated “One of the most common causes of admission in COVID-19 was respiratory failure, however the data on their respiratory status was lacking. Providing and controlling for respiratory factor particularly need for mechanical ventilation, duration of mechanical ventilation, need for tracheostomy, major life sustaining support including dialysis, ECMO are needed to truly needed to better understand if the effect on discharge, readmission, stroke and mortality being seen is completely driving by neurological symptoms or other confounders.” You responded that you do not have statistical power to attribute outcomes to different respiratory factors. However, because of the clinical importance of these variables, the Editors feel strongly that these variables should be included in the univariable analysis along with the other variables considered (noting that there were small numbers with regard to a number of other comorbidities, which would also limit the statistical power).

**Thank you for your detailed comment and suggestion. We agree with the statement that “Providing and controlling for respiratory factor particularly need for mechanical ventilation, duration of mechanical ventilation, need for tracheostomy, major life sustaining support including dialysis, ECMO are needed to truly needed to better understand if the effect on discharge, readmission, stroke and mortality being seen is completely driving by neurological symptoms or other confounders.”**

**However, we believe we have justifiable reasons not to include these variables in the model. During the early chaotic phase of COVID-19 pandemic, many of these life-saving treatments were not consistently applied across patients.** **With overburdened hospital conditions, many general hospital floors, hallways, and ED rooms also became makeshift ICU rooms, resulting in some inaccurate documentation. There could be hypes in the literature but acute ECMO and acute dialysis in acute COVID-19 were rare in our cohort. Given the small sample size, inconsistency of treatments and potential inaccurate documentation, we feel that analysis linking them to various outcomes (discharge, readmission, stroke and mortality) is inappropriate and misleading.**

**R3 suggests that we should include a statement of limitation and cite literature provided. We thus add the following.**

Respiratory parameters such as the need for mechanical ventilation, duration of mechanical ventilation, need for tracheostomy, major life sustaining support including dialysis, extracorporeal membrane oxygenation are needed to better understand if the effect on discharge, readmission, stroke and mortality being seen is completely driving by neurological symptoms or other confounders [51-54]. However, during the early chaotic phase of COVID-19 pandemic, many of these life-saving treatments were not consistently applied across patients. With overburdened hospital conditions, many general hospital floors, hallways, and emergency rooms became makeshift ICU rooms, resulting in some inaccurate documentation. Extracorporeal membrane oxygenation and dialysis during acute COVID-19 were rare in our cohort. Given the small sample size, inconsistency of these respiratory treatments and potential inaccurate documentation of these treatment, analysis to link these treatments to various outcomes (discharge, readmission, stroke, and mortality) could be misleading and was not performed.

    2) Abstract – the Editors feel strongly that the data presented in the paper do not justify the claim to have identified a new syndrome that is distinct from Long COVID. Please remove the following text from the Abstract “…suggest the existence of a novel syndrome of persistent SARS-CoV-2/COVID-19 critical care illness associated with enhanced morbidity and mortality associated with initial neurological involvement that is distinct from Long COVID syndrome. These observations reinforce the notion of dynamic nervous system-systemic crosstalk in SARS-CoV-2/COVID-19 that give rise to a unique “dyshomeostasis syndrome”, with profound implications for a more nuanced perspective on disease pathogenesis and innovative therapeutic initiatives.”

**The text was removed from the abstract as suggested.**

    3) Introduction, lines 144-146. Please remove the statement, “Patients with neurological complications have been shown to have worse acute COVID-19 outcomes including a higher incidence of critical care illness and death compared to propensity-matched controls, thereby defining a novel COVID-19 syndrome distinct from ‘long COVID’”. As stated previously, the Editors do not feel that the published data merit the claim of identifying a new syndrome that is distinct from Long COVID.

**The phrase “thereby defining a novel COVID-19 syndrome distinct from long COVID” was removed from the introduction.**

    4) Abstract and Results: It is unclear why some of the 95% CIs are expressed as percentages. Eg. Lines 332-335: “Mortality rates post COVID-19 hospitalization were also higher in the neurological cohort compared to the control cohort at 0.5 years (OR=2.106, 95% CI=(1.28%, 6.84%); p<0.001), 1 year (OR=1.866, 95% CI=(1.22%, 7.30%), p<0.001), and 3 years (OR=1.787, 95% CI=(2.50%, 9.84%), p<0.001).” Please express these in the conventional way (e.g. “OR:1.222 (95% CI [1.101,1.789; p<0.001”).

**Thank you for catching this error. As suggested in a later comment, this data was presented as N (%) and the 95% CIs were removed from Table 1. We also express other variables in the conventional way as suggested.**

    5) Discussion, line 432-433: The Editors do not feel that the data support the conclusion regarding cause of death, and we ask that this sentence by modified/toned down or omitted: “Of those who died post-discharge, more than half died within the first 0.5 years in both groups, suggesting that most of these deaths were likely COVID-19 related.” Was COVID-19 listed as the primary cause of death for most/all of these patients?

**Upon further review of the data, the majority of the causes of death for these COVID-19 patients who died within 0.5 years after discharge was unknown (31.58%). The second most common cause of death was COVID-19 (17.11%). The phrase “suggesting that most these deaths were likely COVID-19 related” was removed from the discussion as suggested.**

    SPECIFIC POINTS

    DATA AVAILABILITY STATEMENT

    Please update the manuscript online submission form with the details provided about data availability (ll.295-297) in the main manuscript and remove the statement from the main text.

**The data availability statement was removed from the main text and updated in the online submission form as suggested.**

    ABSTRACT

    1) Please define all numerical values (i.e. standard deviation (SD), standard error (SE) etc.). E.g., l.71 “69.71±15.80 years old”.

**The definition “(mean±standard deviation (SD))” was added to the abstract as suggested.**

    2) It is unusual to include ORs and p-values for differences between groups in baseline variables (e.g., sex, race/ethnicity). Please simply report these data as N (%).

**The differences between groups in baseline characteristics are reported simply as N (%). The p-values and ORs have been removed as suggested.**

    AUTHOR SUMMARY

    1) Please define ‘MRI’ and ‘CT’.

**MRI and CT were defined as suggested.**

    2) Please remove the HR from the second bullet point in the “What did the researchers do and find” section.

**The HR was removed from the second bullet point as suggested.**

    INTRODUCTION

    ll.139-141: Please add references to support the following statement, “Additional studies suggest that a diffuse microvasculopathy may ensue with endothelial compromise, micro-infarctions, subsequent microhemorrhages, and microglial conglomerates with innate immune activation.”

**Three references were cited to support the statement as suggested.**

    METHODS

    1) l.191: Please define ‘EMR’ at first use.

**EMR at first use is defined as suggested.**

    2) l.204: Please define all abbreviations at first use (e.g., ICD).

**All abbreviations at first use (ICD-10, SNOMED, LOINC) are defined as suggested.**

    3) Please define all numerical values (i.e. standard deviation (SD), standard error (SE) etc.). E.g., l.306 “672.07±269.76 day” or l.307 “(0.138, 1037.413 days)”. Please revise throughout the entire manuscript.

**Abbreviations were defined for those two circumstances, as well as generally in the Methods section as suggested.**

    4) ll.346-347: The term "trend" is used to refer to a nonsignificant P value. The term trend should be used only when the test for trend has been conducted. Please revise accordingly.

**The term “trend” was removed from the text as suggested.**

    5) ll.352-353: “There were no group differences in cause of death between patients belonging to neurological and control cohorts (all p>0.05).“ – Since we require reporting of exact p-values, we suggest either reporting the exact p-value of each result or, since the results are all not statistically significant, removing "(all p>0.05)" and referring to the tabulated results. In all cases where "(all p>0.05)" is reported, please modify accordingly.

**“All p>0.05” was removed for cause of death and replaced with the exact p-value of each result as suggested. “All p>0.05” was also removed from Table 4A results and replaced with “no statistically significant group differences” since results were not tabulated.**

    6) ll.358-365: If possible/applicable, please report results quantified with 95% CIs as well as p values. At minimum when stating significance, please report p-values.

**The results quantified with p-values have been reported where applicable as suggested.**

    7) ll.372-373: The expression “approaching significance” seems to refer to a nonsignificant P value. As stated previously, please refrain from describing trends (or similar) when the test for trend has not been conducted. Please revise accordingly throughout the entire manuscript.

**Statements referring to “trends” or “approaching significance” are removed from the manuscript as suggested.**

    DISCUSSION

    1) l.403: Please remove the HR.

**The HR was removed as suggested.**

    2) l.424: What does "(16, 20%)“ mean?

**The statement was edited to “16% and 20%” for clarity as suggested.**

    3) l.437: Please exchange the term ‚elderly’ with ‘older’.’

**The term “elderly” was replaced with “older” as suggested.**

    4) Please replace “COVID-19 patients” with “patients with COVID-19” (e.g. l.399). Please revise throughout the entire manuscript.

**“COVID-19 patients” has been replaced with “patients with COVID-19” throughout the entire manuscript as suggested.**

    5) ll.450-451: Please refrain from describing trends (or similar) when the test for trend has not been conducted.

**The term “trending” was removed from the manuscript as suggested.**

    6) l.476: Please temper assertions of primacy ("Our study is novel because […]”) by adding ‘to the best of our knowledge’ or similar.

**“To the best of our knowledge” was added to that sentence as suggested.**

    TABLES AND FIGURES

    1) Table 1: It is unusual to include CI and p-values for differences between groups in baseline variables (e.g., sex, race/ethnicity). Please simply report these data as N (%).

**The column labeled “95% CI of Mean/Proportion Difference” was removed from Table 1 as suggested. The data reported in that column was removed from the manuscript as well.**

    2) Table 3: Please define ‘yo’.

**Changed to “year old” as suggested.**

    3) Supplemental Table: Please report the exact p-values.

**The exact p-values are reported in Supplemental Table 1 as suggested.**

    4) Supplemental Figure 2 (A): Please ensure that the y-axis is identical for (A) to facilitate comparison.

**The y-axis in both charts in Supplemental Figure 2(A) are now identical as suggested.**

    Comments from the reviewers:

    Reviewer #2: Thank you to the authors for addressing my previous comments well. I have no further issues to raise.

**Thank you.**

    Reviewer #3: Thank you so much for providing me with opportunity to review the revised the manuscript.

    I would have preferred to have more information available regarding the hospital course of this patients particularly need for respiratory support, given the retrospective nature, the author mentioned they were unable to obtain it and control for it.

    I would prefer the author to include the following in limitation section.
    The information on their hospital course particularly need for mechanical ventilation, tracheostomy and other life support were not available. Use the following literature to expand on the limitation (PMID: 33329361, PMID: 35994441) Our group also suggested that brain injury is common in ARDS (non-covid and COVID) during their stay --> (PMID: 36094464, PMID: 37667080).  In absence of data on severity of respiratory failure, MV, it is challenging to say that the poor outcome we see is all from neurological symptoms and not from interventions that caused both the neurological symptoms and poor outcome.

**Thank you for the references. We agree with your comments and suggestions to add such limitation. The statement and citations were added to our limitations section as suggested. Please see respond to Editor’s first comment for these edits.**
