# Supplementary material for: Long-term outcomes of hospitalized patients with SARS-CoV-2/COVID-19 with and without neurological involvement: 3-year follow-up assessment
Source: PLoS Med. 2024 Apr 4;21(4):e1004263. doi: 10.1371/journal.pmed.1004263 (PMC10994395; doi:10.1371/journal.pmed.1004263)
Supplement: Attachment — Submitted filename: Response to Reviewers.docx [file pmed.1004263.s009.docx]

**Dear Editor and Reviewers,**

**Please find our point-by-point responses in bold and the revised paper. Thank you.**

Dear Dr. Duong,

I hope this finds you well. Thank you for submitting the revised version of your manuscript (PMEDICINE-D-23-01747). The editorial team has reviewed and discussed your responses to our comments, and we appreciate that you have made the requested changes to the text regarding the identification of a novel syndrome and also regarding causes of death. However, we remain concerned that our comments regarding the inclusion of respiratory factors have not been adequately addressed.

While we acknowledge that the COVID-19 pandemic was a stressful and often chaotic time for hospitals (particularly during the first wave of the pandemic), we feel that the respiratory data in question need to be quantitatively reported in the current manuscript, despite the potential limitations with regard to accuracy and consistency of reporting that you mention. That said, if these data were particularly subject to inaccuracies (relative to other clinical parameters included in the paper, for example), we feel strongly that the circumstances of potentially inaccurate or missing documentation should be described in detail, as they would be interesting and valuable to readers and other researchers.

**Response: Thank you for patience. The potential inaccurate documentation in our previous response referred to makeshift ICU rooms. This has been rectified and this variable are properly documented.**

In addition, we noted that in a previous publication ([https://doi.org/10.1016/j.lana.2021.100041](https://nam04.safelinks.protection.outlook.com/?url=https%3A%2F%2Fdoi.org%2F10.1016%2Fj.lana.2021.100041&data=05%7C02%7Canna.eligulashvili%40einsteinmed.edu%7C1e69260973f549e3f9d508dc024cde31%7C9c01f0fd65e040c089a82dfd51e62025%7C0%7C0%7C638387776478294030%7CUnknown%7CTWFpbGZsb3d8eyJWIjoiMC4wLjAwMDAiLCJQIjoiV2luMzIiLCJBTiI6Ik1haWwiLCJXVCI6Mn0%3D%7C3000%7C%7C%7C&sdata=yb0hhapVvW2ePWkTJCeigiG9WHSQDhDpDmodpLBA%2F6k%3D&reserved=0); Lancet Regional Health - Americas), data on respiratory factors such as mechanical ventilation were available and reported. Given that the data sources used in the two papers are the same, we see no reason why mechanical ventilation cannot be reported in this paper as well. We appreciate that the numbers will be relatively small (particularly when broken down by outcome), so we do not expect you to have sufficient power to do meaningful statistical analyses. But we must insist that you present the data available on respiratory factors at least at a descriptive level (e.g., numbers of patients in each group who had mechanical ventilation in hospital [in main paper], and an indication of the number of patients who had MV in hospital for each outcome [as a supplemental table]).

**Response: ICU and IMV status have been added to Tables 1 and 3A. We also added Supplemental Table 3, which gives the number of patients who were in ICU or IMV for each outcome.**

In light of the data presented in the above-mentioned publication, we would like to encourage you to also report on intensive care unit admission and acute kidney injury, given the association of these variables with subsequent cardiovascular events and death. However, the inclusion of data on ICU admission and AKI would not be a publication requirement given that these data were not requested by reviewers or editors at an earlier stage.

Finally, for the sake of transparency, we ask that you indicate clearly how many of the patients included in this study were also included in previous publications, so that the overlapping nature of the patient cohorts is clear to readers. As such, please indicate whether some and/or all of the patients included in the current study were also reported in the Lancet Regional Health – Americas publication.

**Response: The following has been added to the Methods: Note that this study cohort was a subset of the patients in the previous paper [18].**

I look forward to hearing from you.

Best wishes,

Alex
