# Supplementary material for: Long-term outcomes of hospitalized patients with SARS-CoV-2/COVID-19 with and without neurological involvement: 3-year follow-up assessment
Source: PLoS Med. 2024 Apr 4;21(4):e1004263. doi: 10.1371/journal.pmed.1004263 (PMC10994395; doi:10.1371/journal.pmed.1004263)
Supplement: Attachment — Submitted filename: Response Letter.docx [file pmed.1004263.s010.docx]

Response to Reviewers

Requests from Editors:

    ABSTRACT

    1) l.75: Please remove the age comparison as this variable was included in the propensity matching.

**Done as suggested.**

    2) Please indicate in the Abstract what variables were used in the propensity score matching.

**Done as suggested.**

    3) Please include ICU and IMV status in the Abstract (as done in line 310).

**Done as suggested.**

    4) l.87: Please define ‘CI’ at first use.

**Done as suggested.**

    5) l.91: Please define ‘OR’ at first use.

**Done as suggested.**

    AUTHOR SUMMARY

    The last bullet point under ‘What Do These Findings Mean?’ point should describe the main limitation of the study's methodology.

**Done as suggested. Added “**This study came from a single health system with limited smaple size**.”**

    METHODS AND RESULTS

    1) ll.308-311: Please remove the age comparison as this variable was included in the propensity matching.

**Done as suggested.**

    2) ll.315-317: “Some laboratory data at admission were worse than those at follow-up and laboratory data of the neurological cohort was worse than those of the control cohort.” – We feel this statement is rather vague. Please be more thorough and careful in the presentation of this data. It would be helpful to quantify these statements, e.g., how many of the measured laboratory values were worse at admission than at follow-up, and how many values were worse in the neurological cohort than in the control cohort (and vice versa).

**Done as suggested. Added: “Neurological patients had lower temperature, AST, ALT, CRP, higher mean arterial pressure, DDIM, BUN, glucose, procalcitonin, BNP, TNT, and pulse oximetry at admission, and higher SBP, BUN, Cr, and BNP at follow-up than control patients.”**

    3) ll.326-328: Please briefly describe the results of the number of patients treated in the ICU or with IMV (including numerical values). For example: “A higher proportion of patients in the neurological group who were readmitted to hospital or experienced MACE post-discharge were treated in the ICU or with IMV during their primary hospitalization compared with the control group (16 [6%] of 272 vs 14 [2%] of 728 for readmission; 5 [6%] of 85 vs 3 [2%] of 198 for MACE; table S3]” (or similar). Please note that we are not sure if we have interpreted the data correctly here (We would like you to present the following data: Of XXX who were readmitted post-COVID, xx (%) had been in the ICU and xx (%) had had IMV).

**Done as suggested. Added: Of 414 neurological and 1043 control patients who were readmitted post-COVID, 30 (7.25%) neurological and 77 (7.38%) control had been in the ICU and 24 (5.80%) neurological and 59 (5.66%) control had IMV.**

    4) ll.351-354: “Both within the neurological and control cohorts, non-survivors were significantly older (neuro: 76.40±11.71 vs 67.85±15.85, p<0.001 and control: 76.56±11.23 vs 69.20±14.95, p<0.001) and had higher COVID-19 severity score (control: 4.74±2.05 vs 3.36±2.06, p<0.001) compared to survivors.” – Please revise the sentence as it currently reads that within the neurological cohort, non-survivors also had a higher COVID-19 severity score compared to survivors, which was not the case (p=0.088). We recommend breaking this into two sentences to avoid confusion.

**Done as suggested.**

    5) ll.370-371: Please change to: "The number of patients who underwent imaging varied between pre-, intra-, and post-COVID-19."

**Done as suggested.**

    DISCUSSION

    1) ll.400-401: “the primary causes of death after discharge for both cohorts were heart failure, sepsis, influenza and pneumonia, COVID-19 and ARDS” – The primary cause of death after discharge was “unknown” – please revise accordingly.

**Done as suggested.**

    2) ll.401-403: “patients who died post-discharge were significantly older, more likely to be of male sex, had higher COVID-19 severity score, and those were sent to skilled nursing facilities at discharge compared to survivors” – The results for sex were not significant. We feel that describing patients who died after discharge as more likely to be male may be misleading. Similarly, only for the control cohort was it true that patients who died post-charge had a higher COVID-19 severity score, which should be clarified here.

**Done as suggested.**

    3) ll.402-403, please change to: “…and were more likely to have been discharged to skilled nursing facilities at discharge…”

**Done as suggested.**

    4) ll.430-431: “...older and more likely to be of male sex as compared to survivors.” – Please clarify that this was not a significant finding.

**Thank you for catching this. We checked and confirmed it is not a significant finding. The text has been revised accordingly.**

    5) ll.436-437: “Here we reported male sex also had worse long-term outcomes post COVID-19 discharge.” – This statement is not supported by the results, therefore please remove or re-phrase accordingly.

**The statement was removed as suggested.**

    6) l.441: “Patients who had more severe COVID-19 disease were also more likely to die post-discharge.” – Please clarify that this only applies to the control cohort.

**Done as suggested.**

    7) ll.486-493 “Moreover, multiple communication routes between the central...including proteotoxicity and protean epigenetic derangements.” – please provide references.

**Done as suggested.**

    TABLES AND FIGURES

    1) Table 4A: Please define ‘NA’. Please include dates (or time frames) for Pre, During, and Post COVID-19.

**Done as suggested.**

    2) Table 4B: Please include dates (or time frames) for Pre, During, and Post COVID-19.

**Done as suggested.**

    3) Figure 1: In the figure description, please define ‘Other’.

**Done as suggested.**

    4) Supplemental Table 2: Please remove the definition of the p-values from the figure description as you have included the exact values.

**Done as suggested.**

    5) Please ensure to include Supplemental Table 3 in the Supporting Information file.

**Done as suggested.**

    REFERENCES

    We noted that some of the references are not correctly formatted (e.g. [37]). Please thoroughly revise all references and ensure that they display first six authors, et al.

**Done as suggested.**

    SOCIAL MEDIA

    To help us extend the reach of your research, please provide any X (formerly known as Twitter) handle(s) that would be appropriate to tag, including your own, your coauthors’, your institution, funder, or lab. Please respond to this email with any handles you wish to be included when we tweet this paper.

**Please tag @einsteinmed @duongmri @davidaltschulmd**

    Comments from Reviewers:

    Reviewer #2: I've looked over the new results added and the changes to the paper look good. However I couldn't see Supplemental Table 3 in the provided document - perhaps the previous one was included?

**Thank you. Supplemental Table 3 has been added to the Supporting Information file.**

    One minor comment: Line 359: add 'no significant differences'.

**Done as suggested.**

    Any attachments provided with reviews can be seen via the following link:


    ------------------------------------------------------------

    General Editorial Requests

    1) We ask every co-author listed on the manuscript to fill in a contributing author statement. If any of the co-authors have not filled in the statement, we will remind them to do so when the paper is revised. If all statements are not completed in a timely fashion this could hold up the re-review process. Should there be a problem getting one of your co-authors to fill in a statement we will be in contact. YOU MUST NOT ADD OR REMOVE AUTHORS UNLESS YOU HAVE ALERTED THE EDITOR HANDLING THE MANUSCRIPT TO THE CHANGE AND THEY SPECIFICALLY HAVE AGREED TO IT.

**Done as suggested on Page 2 on the manuscript.**

    2) Please ensure that the paper adheres to the PLOS Data Availability Policy (see <https://nam04.safelinks.protection.outlook.com/?url=http%3A%2F%2Fjournals.plos.org%2Fplosmedicine%2Fs%2Fdata-availability&data=05%7C02%7Canna.eligulashvili%40einsteinmed.edu%7Ca5761f962f694e30970b08dc2eea21ea%7C9c01f0fd65e040c089a82dfd51e62025%7C0%7C0%7C638436830328451157%7CUnknown%7CTWFpbGZsb3d8eyJWIjoiMC4wLjAwMDAiLCJQIjoiV2luMzIiLCJBTiI6Ik1haWwiLCJXVCI6Mn0%3D%7C0%7C%7C%7C&sdata=ddQC612N1KUNqVRKJaMdGxScj0Y8umaZG%2FzZo65uA0w%3D&reserved=0)>, which requires that all data underlying the study's findings be provided in a repository or as Supporting Information. For data residing with a third party, authors are required to provide instructions with contact information for obtaining the data. PLOS journals do not allow statements supported by "data not shown" or "unpublished results." For such statements, authors must provide supporting data or cite public sources that include it.

**Data availability statement with link to repository has been added to manuscript.**

    3) Please review your reference list to ensure that it is complete and correct. If you have cited papers that have been retracted, please include the rationale for doing so in the manuscript text, or remove these references and replace them with relevant current references. Any changes to the reference list should be mentioned in the rebuttal letter that accompanies your revised manuscript.
